# Integrative Approaches in Structural Science: Bridging Electron and X-ray Techniques

**DOI:** 10.1063/4.0000848

**Published:** 2025-10-27

**Authors:** Brandon Q. Mercado

**Affiliations:** Yale University, New Haven, Connecticut, USA

## Abstract

Chemistry core facilities have long hosted multimodal instrumentation alongside specialized systems designed for targeted scientific inquiries. At Yale University's Chemical and Biophysical Instrumentation Center, we have strategically consolidated resources for scattering, imaging, and diffraction into a unified Structural Science Facility. My presentation will focus on the integration of complementary structural determination techniques, specifically MicroED, single-crystal X-ray diffraction, powder X-ray diffraction, and SAXSWAXS, within a cohesive ecosystem of university cores, highlighting how such consolidation enhances their collective scientific capabilities. Additionally, I'll discuss how this integrative philosophy extends to collaborations with partner cores. I will highlight use cases, successful workflows, and best practices for maintaining effective interoperability among these complementary methods.

